# Supplementary material for: Multifaceted regulation of siderophore synthesis by multiple regulatory systems in Shewanella oneidensis
Source: Commun Biol. 2024 Apr 25;7:498. doi: 10.1038/s42003-024-06193-7 (PMC11045786; doi:10.1038/s42003-024-06193-7)
Supplement: Supplementary file 2 — Supplementary information [file 42003_2024_6193_MOESM2_ESM.pdf]

Supporting Information for

Multifaceted regulation of siderophore synthesis by multiple regulatory systems in *Shewanella oneidensis*

Peilu Xie,† Yuanyou Xu,† Jiaxin Tang, Shihua Wu,* and Haichun Gao*

*Corresponding authors: Shihua Wu (drwushihua@zju.edu.cn) and Haichun Gao (haichung@zju.edu.cn)

This PDF file includes:

- Supplementary Figures 1 to 8
- Supplementary Tables 1 to 3
- Description of supplementary Movie 1
- Supplementary data (uncropped blots)

Other supporting materials for this manuscript include the following:

- Supplementary Movie 1

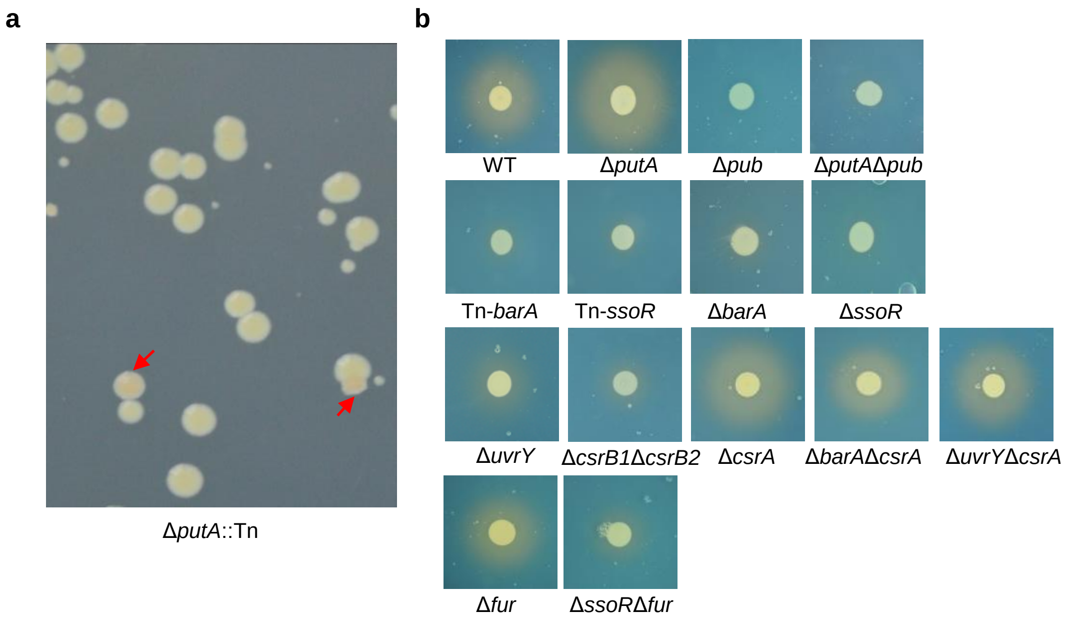

Supplementary Figure 1. Screening for suppressor mutants of Δ*putA* and verification. a Transposon mutagenesis screening. A transposon library of Δ*putA* was constructed and spread on LB agar plates for screening colonies that regained reddish-brown color. **b** Siderophore production of indicated *S. oneidensis* strains by CAS agar assay. Cultures (OD_{600} of ~0.6) of indicated strains were adjusted to the same OD, from which 10 μl was dropped on LB agar plates and incubated for certain time when growth was comparable (judged by the diameters of the visible cell drops), followed by pouring in CAS reagent to completely cover the entire plates. The formation of chelated halos was observed and photographed after three hours.

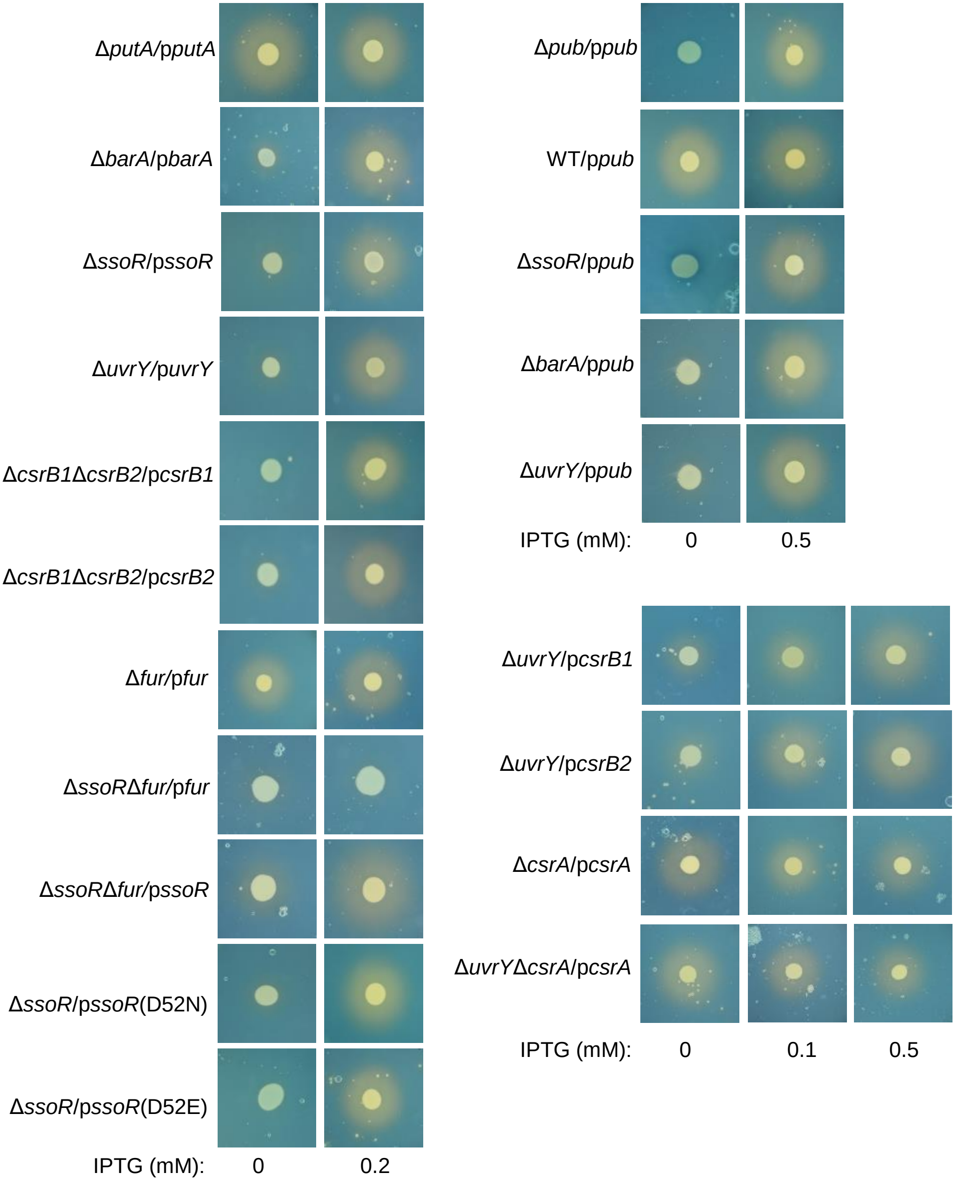

Supplementary Figure 2. The siderophore production of mutants and their complementary strains used in the study. Expression of genes was driven by IPTG-inducible promoter P*tac* with IPTG at varying concentrations.

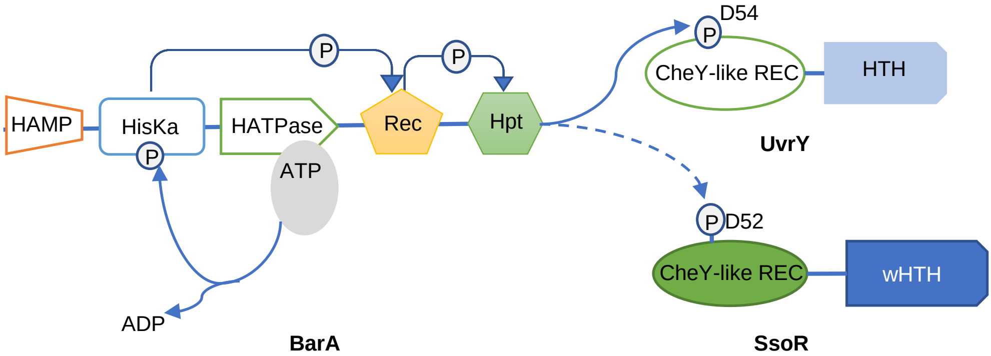

Supplementary Figure 3. BarA-UvrY TCS and SsoR. BarA is known to phosphorylate UvrY via phospho-relay shown by arrowed lines. BarA may also possibly phosphorylate SsoR by arrowed dash line. HAMP, HAMP signal transduction domain; HisKa, histidine kinase dimerization domain; HATPase, histidine kinase ATPase domain; Rec, receiver domain; Hpt, histidine-containing phosphotransfer domain; wHTH, winged helix-turn-helix DNA-binding motif; HTH, helix-turn-helix DNA-binding motif. D52/54: 4-aspartylphosphate.

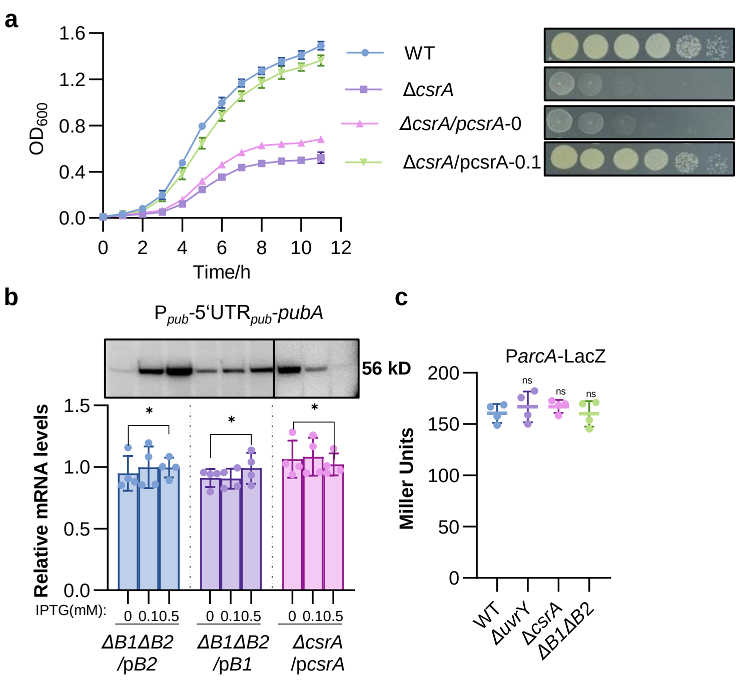

Supplementary Figure 4. The regulation of BarA/UvrY/Csr system on siderophore synthesis. a The growth of *csrA* strains in LB liquid and agar plates. Expression of *csrA* was driven by IPTG-inducible promoter Ptac with 0.1 mM IPTG. **b** The mRNA level of *pubA* and protein level in indicated strains carrying *csr* genes (under the control of *tac* promoters) were respectively measured. **c** The activity of *arcA* promoters are constitutive in the indicated strains.

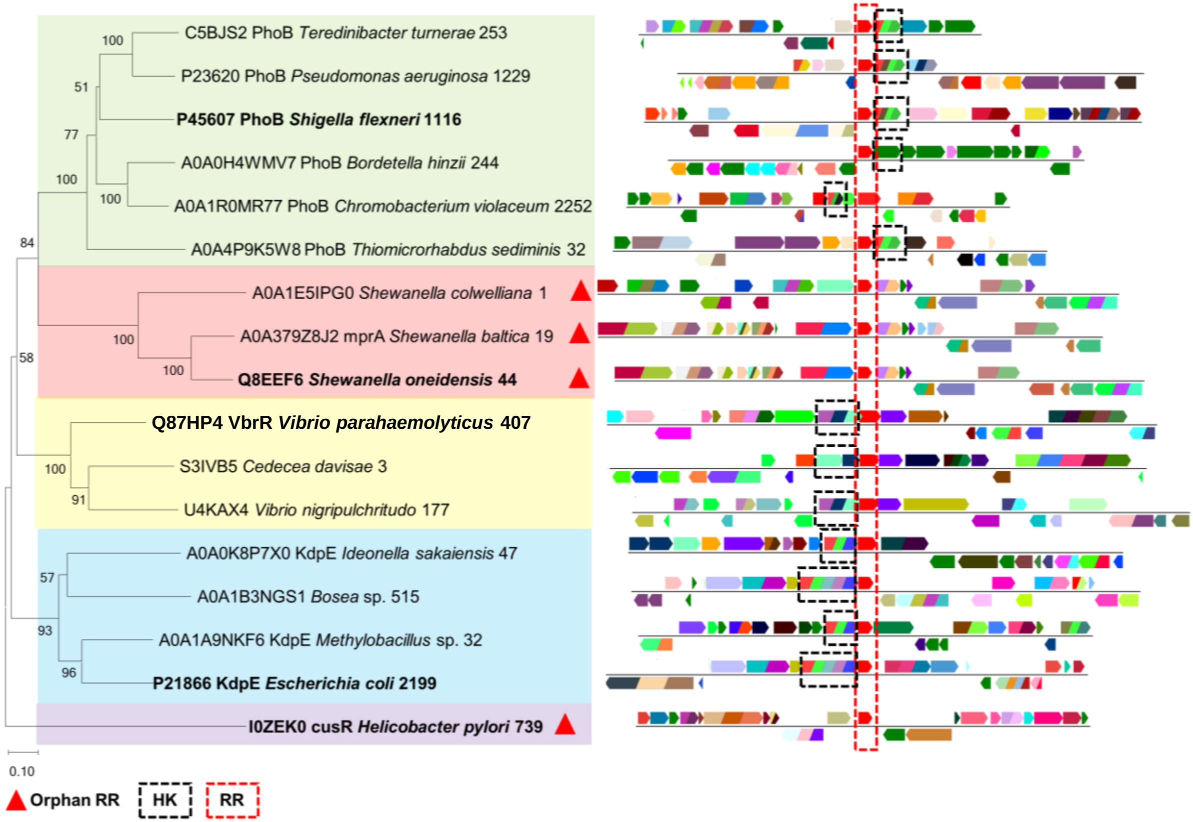

Supplementary Figure 5. Phylogenetic tree and genomic backgrounds of RRs. On left, the phylogenetic tree shown in Figure 5A is given with the full name of the bacterial species. On right, the genomic backgrounds of the genes of interest, with RR genes in red boxes and kinase genes in black boxes.

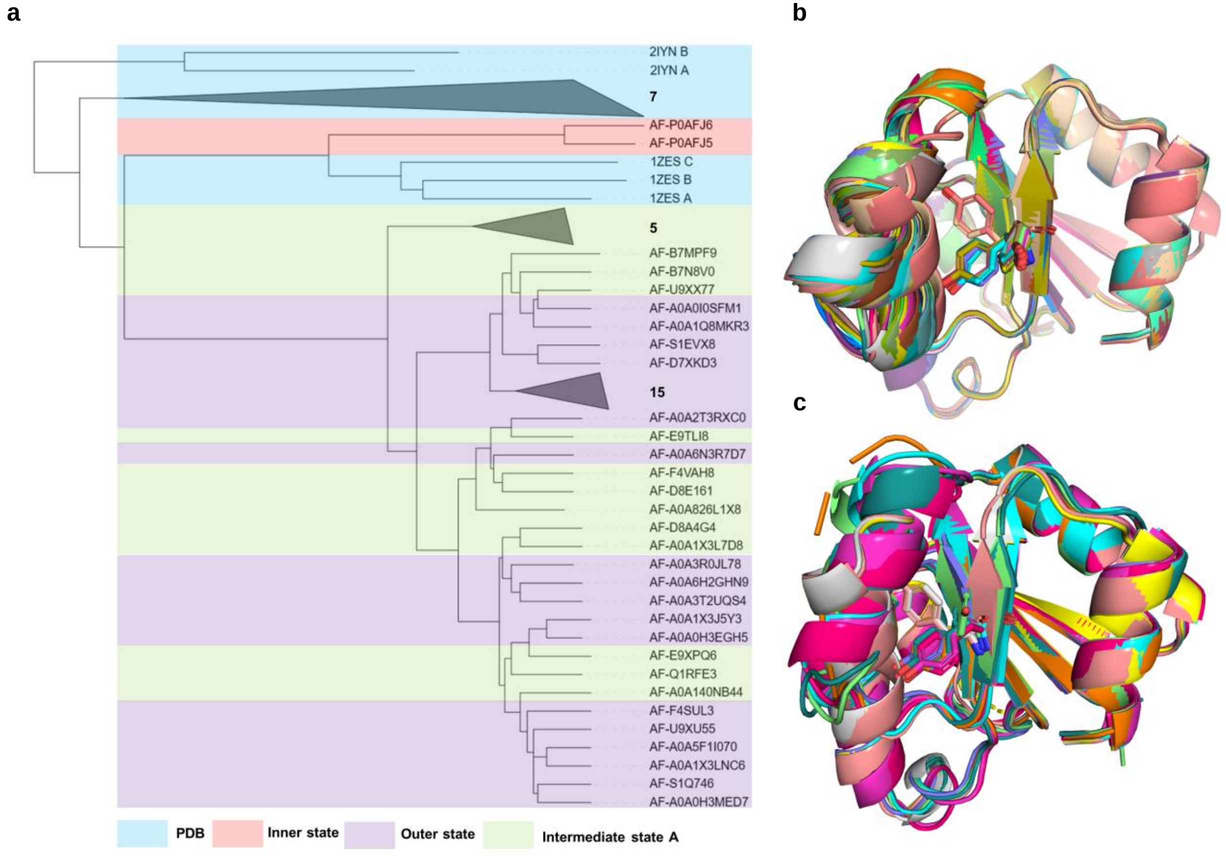

Supplementary Figure 6. Structural similarity dendrogram and alignment results for the AlphaFold-predicted and Crystal structures of *E. coli* PhoBs. a The dendrogram is calculated using DALI and derived by average linkage clustering of the structural similarity matrix (Dali Z-scores). The REC domains on the dendrogram include 12 monomers from five crystal structures of *E. coli* PhoB (highlighted in blue), as well as 52 monomers (with protein sequences identical to *E. coli* PhoB) from the AlphaFold Protein Structure Database. The proteins from the AlphaFold Protein Structure Database are distinguished by the states using red, purple, and green background. **b** and **c** show alignment results for the AlphaFold-predicted structure and Crystal structures, respectively.

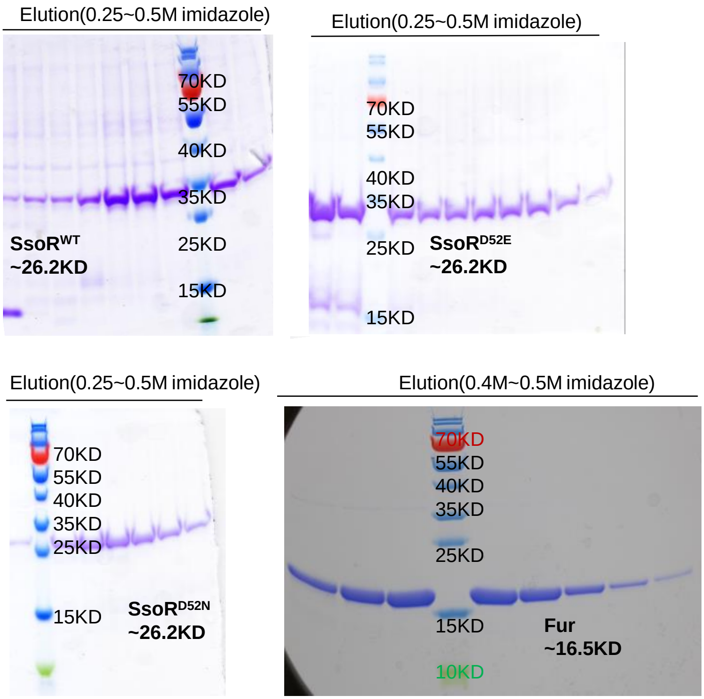

Supplementary Figure 7. Expression and purification of proteins. His_6-tagged recombinant proteins were expressed and purified from *E. coli* BL21(DE3). The purification conditions for Fur protein are natural, whereas the purification buffers for SsoR proteins are all denaturant-added. After induction with 0.2 mM IPTG overnight, cells were collected, disrupted by a French pressure cell disrupter, and debris was removed by centrifugation. The supernatant solution was loaded on a 5-ml HisTrap HP, and fractions were examined by SDS-PAGE and Coomassie brilliant blue staining. To renature the SsoR proteins, the eluted fractions containing SsoR proteins were diluted into 2 M urea, 20 mM Tris/HCl (pH 7.0), 1 mM EDTA by sequential dilutions and then dialyzed against 20 mM Tris/HCl (pH 7.0) overnight.

Fig. 2a

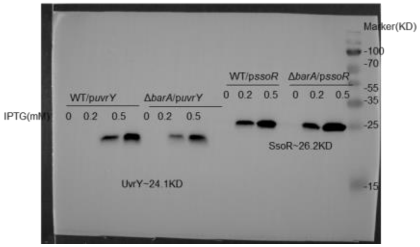

Fig. 2b

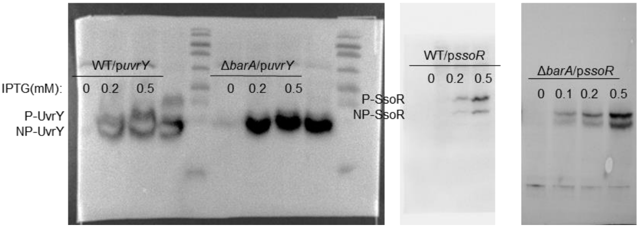

Fig. 3d

Fig. 3e up panel

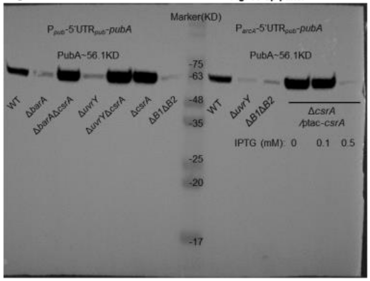

Fig. 3e bottom panel

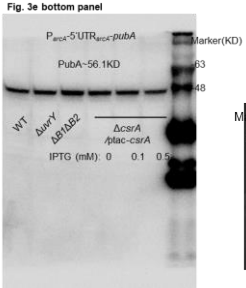

Fig. 6a up panel

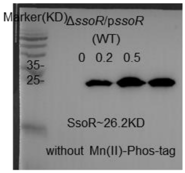

Fig. 6a up panel

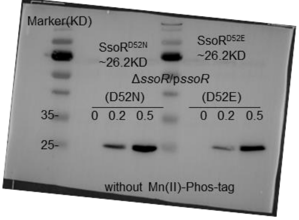

Fig. 4a

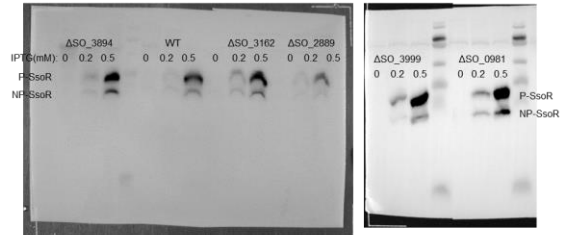

Fig. 6a bottom panel

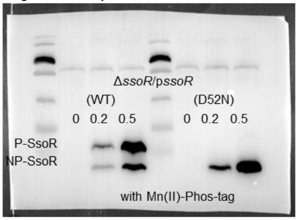

Fig. 6a bottom panel

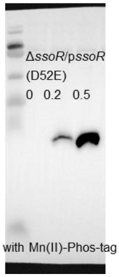

Fig. 8b

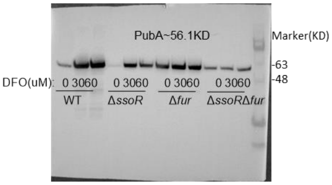

Fig. 8d

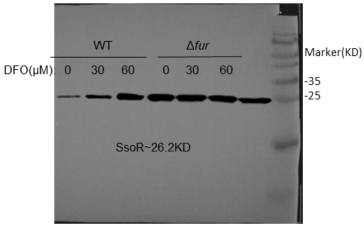

Supplementary Fig. 4b

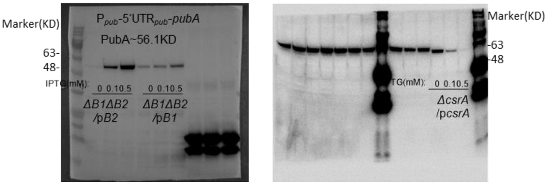

Supplementary Figure 8. All uncropped blots

Supplementary Table 1: TCSs in *S. oneidensis*

Locus	TCSs (HK/RR)	Annotation	Type	Function &
SO_0059	KdpD/KdpE	transcriptional regulatory protein KdpE	OmpR	
SO_0060		sensor histidine kinase kdpD	Classic	
SO_0351	SO_0352/SO_0351	LuxR family DNA-binding response regulator	NarL	
SO_0352		sensor histidine kinase	Classic	
SO_0544	SO_0544/SO_0545	sensory box histidine kinase	Classic	
SO_0545		response regulator	PleD_VieA	
SO_0577	ArcS and HptA/arcA	sensory box histidine kinase ArcS	Hybrid	aerobic respiration control[1]
SO_1327		sensor histidine kinase-like protein HptA	HPt	
SO_3988		two-component response regulator ArcA	OmpR	
SO_0621	SO_0621/SO_0622	sensor histidine kinase	Classic	
SO_0622		DNA-binding response regulator	OmpR	
SO_0859	SO_0859/SO_0860	sensory box histidine kinase	Unorthodox	
SO_0860		response regulator	RpfG	
SO_1228	TorS/ TorR	DNA-binding transcriptional regulator TorR	OmpR	TMAO respiration[2]
SO_1230		sensor histidine kinase TorS	Unorthodox	
SO_1416	SO_1417/SO_1416	DNA-binding response regulator	NarL	
SO_1417		sensor histidine kinase	Classic	
SO_1558	PhoR/PhoB	phosphate regulon response regulator PhoB	OmpR	phosphate transport
SO_1559		phosphate regulon sensor protein PhoR	Classic	
SO_3457	BarA/UvrY	hybrid sensory histidine kinase BarA	Unorthodox	central carbon metabolism[3]
SO_1860		response regulator UvrY	NarL	
SO_1945	PhoQ/PhoP	sensor protein PhoQ	Classic	acid tolerance[4]
SO_1946		transcriptional regulatory protein PhoP	OmpR	
SO_2104	SO_2105/SO_2104	DNA-binding response regulator YgiX	OmpR	
SO_2105		sensor protein YgiY	Classic	
SO_2119	CrsR/CrsA	response regulator CrsR	Hybrid	general stress[5,6]
SO_2120		chemotaxis protein CheY	CheY	
SO_2121		chemotaxis protein CheA	CheA	
SO_2126		protein-glutamate methylesterase CheB	CheB	
SO_2192	PghK/PghR	sensor histidine kinase PghK	Classic	peptidoglycan stress[7]
SO_2193		DNA-binding response regulator PghR	OmpR	
SO_2145	HnoK/HnoB and HnoC and HnoD	sensor histidine kinase HnoK	Classic	NO-responsive multicomponent c-di-GMP signaling network[8-10]
SO_2538		response regulator	RpfG	
SO_2539		response regulator HnoB	VieA	
SO_2540		response regulator HnoC	unclassified	
SO_2541		response regulator HnoD	unclassified	
SO_2543		sensor histidine kinase HnoE	Classic	
SO_2544	HnoS and HnoT /SO_2457	sensor histidine kinase HnoS	Unorthodox	
SO_2545		sensor histidine kinase HnoT	Classic	
SO_2547		response regulator	CheY	
SO_2648	SO_2742/SO_2648	response regulator	NarL	acetyl-CoA synthase[11]
SO_2742		sensor histidine kinase	Hybrid	
SO_2822	SO_2822/SO_2823	sensor histidine kinase	Classic	
SO_2823		response regulator YehT	LytTR	
SO_3206	SO_3207/SO_3206 and SO_3209	chemotaxis-specific methylesterase	CheB	
SO_3207		chemotaxis protein	CheA	
SO_3209		chemotaxis protein CheY	CheY	
SO_3230	FlrB/FlrC	flagellar regulatory protein C FlrC	NtrC	flagellar assembly[12]
SO_3231		flagellar regulatory protein B FlrB	Classic	
SO_3305	SO_3306/SO_3305	LuxR family DNA-binding response regulator	NarL	
SO_3306		sensor histidine kinase	Classic	
SO_3594	RstA/RstB	transcriptional regulator RstA	OmpR	
SO_3595		sensor protein RstB	Classic	
SO_3688	SO_3688/SO_3689	nitrogen regulation protein NtrY	Classic	
SO_3689		σ^{54} dependent nitrogen response regulator	PrrA	
SO_3981	NarQ/NarP	nitrate/nitrite sensor protein NarQ	Classic	Nitrate/nitrite respiration[13]
SO_3982		DNA-binding response regulator NarP	NarL	
SO_4001	SO_4002/SO_4001 and SO_4003	chemotaxis protein CheY	FrzZ	
SO_4002		sensory transduction histidine kinase	Unorthodox	

SO_4003		response regulator	RpfG	
SO_4155	TtrS/TtrR	sensor histidine kinase TtrS	Classic	thiosulfate respiration[14]
SO_4157		DNA-binding response regulator TtrR	NarL	
SO_4172	PrrA/PrrB	DNA-binding response regulator	PrrA	oxygen response [15]
SO_4173		sensor histidine kinase	Classic	
SO_4387	SO_4387/SO_4388	sensor histidine kinase	Classic	pH stress response[16]
SO_4388		DNA-binding response regulator	OmpR	
SO_4427	SO_4427/SO_4428	sensor histidine kinase	Classic	
SO_4428		DNA-binding response regulator	OmpR	
SO_4444	SO_4445/SO_4444	capsular synthesis regulator component B	NarL	
SO_4445		sensor histidine kinase	Unorthodox	
SO_4471	NtrB/NtrC	nitrogen regulation protein NtrB	Classic	nitrogen metabolism[17]
SO_4472		nitrogen regulation protein NtrC	NtrC	
SO_4477	CpxA/CpxR	transcriptional regulatory protein CpxR	OmpR	periplasmic stress[18]
SO_4478		sensor protein CpxA	Classic	
SO_4487	SO_4488/SO_4487	DNA-binding response regulator	OmpR	
SO_4488		sensor histidine kinase	Classic	
SO_4622	SO_4622/SO_4633	sensor histidine kinase	Classic	
SO_4623		DNA-binding response regulator	OmpR	
SO_4633	EnvZ/OmpR	osmolarity response regulator OmpR	OmpR	osmotic stress response[19]
SO_4634		osmolarity sensor protein EnvZ	Classic	
SO_4637	SO_4638/SO_4637	DNA-binding response regulator	OmpR	
SO_4638		sensor histidine kinase	Classic	
SO_4647	SO_4648/SO_4647	DNA-binding response regulator	OmpR	
SO_4648		sensor histidine kinase	Classic	
SO_4717	SO_4717/SO_4718	sensor histidine kinase	Classic	
SO_4718		σ^{54} dependent response regulator	NtrC	
SO_0549	**orphan RRs**	chemotaxis protein CheY	FrzZ	
SO_0570		response regulator	unclassified	
SO_1989		chemotaxis protein CheV	CheV	
SO_2127		response regulator	PleD	
SO_2318		chemotaxis protein CheY	CheY	
SO_2327		chemotaxis-specific methylesterase	CheB	
SO_2366		response regulator	RpfG	
SO_2426		DNA-binding response regulator	OmpR	
SO_3123		chemotaxis protein CheV	CheV	
SO_3138		C4-dicarboxylate transport regulator DctD	NtrC	
SO_3196		response regulator	unclassified	
SO_3252		chemotaxis protein CheV	CheV	
SO_0981	**orphan HKs**	sensor histidine kinase	HisKa	
SO_2889		sensory box histidine kinase	Classic	
SO_3162		sensor histidine kinase	Classic	
SO_3894		sensor histidine kinase	Classic	
SO_3999		sensor histidine kinase	Classic	

Supplementary Table 2: Strains and plasmids used in this study

Strain or plasmid	Description	Reference or
E. coli strains		
DH5α	Host for cloning	Laboratory stock
WM3064	Δ*dapA*,donor strain for conjugation	W.Metcalf, UIUC
BL21(DE3)	Recombinant protein expression host strain	Laboratory stock
S. oneidensis strains		
MR-1	Wild type	Laboratory stock
HG3030-2	Δ*pub* mutant derived from MR-1	20
HG3033	Δ*putA* mutant derived from MR-1	20
HG3033-33	Δ*putA*Δ*pub* mutant derived from MR-1	20
Tn-SsoR	Suppressor derived from Δ*putA*	This study
Tn-*barA*	Suppressor derived from Δ*putA*	This study
HG2426	Δ*ssoR* derived from MR-1	This study
HG3457	Δ*barA* derived from MR-1	This study
HG1860	Δ*uvrY* derived from MR-1	This study
HG3426	Δ*csrA* derived from MR-1	This study
HG3162	ΔSO_3162 derived from MR-1	This study
HG2889	ΔSO_2889 derived from MR-1	This study
HG3894	ΔSO_3894derived from MR-1	This study
HG3999	ΔSO_3999 derived from MR-1	This study
HG0981	ΔSO_0981 derived from MR-1	This study
HGcsrB	Δ*csrB1*Δ*csrB2* derived from MR-1	This study
HG3457-3426	Δ*barA*Δ*csrA* derived from MR-1	This study
HG1860-3426	Δ*uvrY*Δ*csrA* derived from MR-1	This study
HG1937	Δ*fur* mutant derived from MR-1	21
HG1937-2426	Δ*ssoR*Δ*fur* mutant derived from MR-1	This study
Plasmids		
pHGM01	Att-based suicide vector, Ap^r, Gm^r, Cm^r	22
pHGEI01	Km^r, integrative *lacZ* reporter vector	23
pHGEN-Ptac	Km^r, IPTG-inducible expression vector	24
pHG101	Km^r, promoterless broad-host vector	25
pFAC	Gm^r, vector containing transposable sequence	Laboratory stock
pET-28a(+)	His-tagged protein expression vector, Ap^r,Km^r	Novagen
pHGE-P*tac-pubABC*	Vector for inducible expression of *pubABC*	This study
pHGE-P*tac-ssoR*	Vector for inducible expression of His_6-tagged *SsoR*	This study
pHGE-P*tac-ssoR*$^{(D52N)}$	Vector for inducible expression of His_6-tagged SsoR$^{(D52N)}$	This study
pHGE-P*tac-ssoR*$^{(D52E)}$	Vector for inducible expression of His_6-tagged SsoR$^{(D52E)}$	This study
pHGE-P*tac-barA*	Vector for inducible expression of *BarA*	This study
pHGE-P*tac-uvrY*	Vector for inducible expression of His_6-tagged *UvrY*	This study
pHGE-P*tac-csrA*	Vector for inducible expression of *CsrA*	This study
pHGE-P*tac-csrB1*	Vector for inducible expression of CsrB1	This study
pHGE-P*tac-csrB1*	Vector for inducible expression of CsrB2	This study
pHG101-*pubA*^{his}	pHG101 containing promoter of *pub* and 5'UTR and CDS of *pubA*	This study
pHG101-P$_{arcA}$-5'UTR$_{pub}$-*pubA*^{his}	pHG101 containing promoter of *arcA* and 5'UTR and CDS of *pubA*	This study
pHG101-P$_{arcA}$-5'UTR$_{arcA}$ -*pubA*^{his}	pHG101 containing promoter and 5'UTR of *arcA* and CDS of *pubA*	This study
pHG101-*ssoR*^{his}	pHG101 containing leader region and CDS of *ssoR*	This study
pHGEI01-P$_{ssoR}$	P$_{ssoR}$-*lacZ* fusion within pHGEI01	This study
pHGEI01-P$_{arcA}$	P$_{arcA}$-*lacZ* fusion within pHGEI01	This study
pHGEI01-P$_{pub}$	P$_{pub}$-*lacZ* fusion within pHGEI01	This study
pET-28a(+)-*fur*	pET-28a(+) expressing His_6-tagged Fur	21
pET-28a(+)-*ssoR*	pET-28a(+) expressing His_6-tagged SsoR	This study
pET-28a(+)-*ssoR*$^{(D52N)}$	pET-28a(+) expressing His_6-tagged SsoR$^{(D52N)}$	This study
pET-28a(+)-*ssoR*$^{(D52E)}$	pET-28a(+) expressing His_6-tagged SsoR$^{(D52E)}$	This study

Supplementary Table 3: Primers used in this study

Primers	Sequences
In-frame deletion	
ssoR-5O	GGGGACAAGTTTGTACAAAAAAGCAGGCTGCGGTATCGACTTCAACTTA
ssoR-3O	GGGGACCACTTTGTACAAGAAAGCTGGGTTGGATTGCCATCACCGACTTT
ssoR-5I	CGCATCAGTGCAATGGATTACTAAGCACCAAACGAGAATC
ssoR-3I	TAATCCATTGCACTGATGCGCAAGGATACAGTTTTAGCG
barA-5O	GGGGACAAGTTTGTACAAAAAAGCAGGCTAAATCAGACAAAGGCTTAGC
barA-3O	GGGGACCACTTTGTACAAGAAAGCTGGGTAAGATTGGTTTGAATCGCCCC
barA-5I	GCCTGCCATCAATTCGATTATAAGTTCTAGGGACGATGAG
barA-3I	TAATCGAATTGATGGCAGGCAGCTAACCATAAGTCGGGC
uvrY-5O	GGGGACAAGTTTGTACAAAAAAGCAGGCTGGCGCCAGCAAATTTTGCCAT
uvrY-3O	GGGGACCACTTTGTACAAGAAAGCTGGGTCGAAGACATAGTCATCTAAGGCGT
uvrY-5I	TCCATCAGAAGACAGTGTGAAGCCGCAGCGCTGTTTCCTAAG
uvrY-3I	TCACACTGTCTTCTGATGGAGCGTTTAAAAGCGCTAGAACT
csrA-5O	GGGGACAAGTTTGTACAAAAAAGCAGGCT GAAGGTTTGTTCACCAGCAG
csrA-3O	GGGGACCACTTTGTACAAGAAAGCTGGGTCATAACTGATGCAGAAATGGTGC
csrA-5I	TCTAGGTATTTACAGAGCGGGCATTCGATAGCTCCTTTATGTCT
csrA-3I	CCGCTCTGTAAATACCTAGATACAAAGCTTATGCGCGTATTAGC
csrB1-5O	GGGGACAAGTTTGTACAAAAAAGCAGGCTGTTATCGCTCTTTCGCTGAGT
csrB1-3O	GGGGACCACTTTGTACAAGAAAGCTGGGTGCTTTAAGTTCGGGGATGAGTTT
csrB1-5I	TCTAGGTATTTACAGAGCGGAATTCTACGTGAATCCCGCCCT
csrB1-3I	CCGCTCTGTAAATACCTAGACTTACAGAGGATAAATGGGGCG
csrB2-5O	GGGGACAAGTTTGTACAAAAAAGCAGGCTTAACTATACCGCACGGACTTTG
csrB2-3O	GGGGACCACTTTGTACAAGAAAGCTGGGTTCACTTATTGGGTGAATTACGCAA
csrB2-5I	TCTAGGTATTTACAGAGCGGTGAAGTCTACGACAGGCATGATAA
csrB2-3I	CCGCTCTGTAAATACCTAGATGTATGGGGAATTTAACTTCGGTAG
SO_3162-5O	GGGGACAAGTTTGTACAAAAAAGCAGGCTTAGCGTGGTTTGAATACCTTC
SO_3162-3O	GGGGACCACTTTGTACAAGAAAGCTGGGTGAGCGACTGTAGTTATCTTGT
SO_3162-5I	GGCGGCTGCCAACATAACAAGCGTCATAACGATGCTATTCC
SO_3162-3I	TTGTTATGTTGGCAGCCGCCACCAGAATTTAGAAAGCTCGC
SO_2889-5O	GGGGACAAGTTTGTACAAAAAAGCAGGCT CCTAACGTCGCCTTGGTATC
SO_2889-3O	GGGGACCACTTTGTACAAGAAAGCTGGGTCATTTATGTTGCCTTGTCCTGTC
SO_2889-5I	TCTAGGTATTTACAGAGCGG CTCAATGTTAGGCCGTTTATCCAT
SO_2889-3I	CCGCTCTGTAAATACCTAGATTGGCCGCTATCTAAGGCTTA
SO_3894-5O	GGGGACAAGTTTGTACAAAAAAGCAGGCTGTTCGGGGACAAACTTATCA
SO_3894-3O	GGGGACCACTTTGTACAAGAAAGCTGGGTGGCGCTTATTGCTTGGTGTA
SO_3894-5I	TTAAGGCTACGAGACTTTGGCCTGCCAATCGGACTTTTTA
SO_3894-3I	CCAAAGTCTCGTAGCCTTAATATCCCCCTAAATCGATGCG
SO_3999-5O	GGGGACAAGTTTGTACAAAAAAGCAGGCTGCATGCCACTGATAAACAGC
SO_3999-3O	GGGGACCACTTTGTACAAGAAAGCTGGGTGATTGTGGCGATTTCGTCT
SO_3999-5I	CCTGCGTGCAAATCTAACAACGCTGAAGAATCCCCTGATG
SO_3999-3I	TTGTTAGATTTGCACGCAGGTGTTTACCCGCAGCAACTT
SO_0981-5O	GGGGACAAGTTTGTACAAAAAAGCAGGCT CTTCACCATTTCACGATTCCCT
SO_0981-3O	GGGGACCACTTTGTACAAGAAAGCTGGGT GGCGAATTTGCACCAAAGTC
SO_0981-5I	TCTAGGTATTTACAGAGCGG AAGGGGGTGTTCTGGTCTAAT
SO_0981-3I	CCGCTCTGTAAATACCTAGA GGGATGATAGGTTATTGCCTAAAGC
Complementation	
pub-CF	GTCGACTTTTATCGCTGACTCGCAGT
pub-CR	CTCGAGTTAGGGTTTTAGCTTCATATTTGCTGAGCCAAAGGCA
ssoR-CF	GAATTCATGAGTAGAATACTGTTAGTCG
ssoR-CR	AAGCTTGGTGAATCGACTCACAGAGG
barA-CF	GAATTCATGAACCCTGTCAACAAC
barA--CR	AAGCTTAACGCTTAAAGAAGCCCG
uvrY-CF	GAATTCCAGCATTGGTTAAAGGTGGA
uvrY-CR	AAGCTTCTCACGCCTAACACACTATA
csrA-CF	CCGGAATTC ATGCTGATTTTGACTCGTCGT
csrA-CR	CCC CTCGAG TTAAAAGTTACCGCCTTCAGAAGG
csrB1-CF	CCGGAATTC TAAATGGATAGCACGGATAGCAAT
csrB1-CR	CCC CTCGAG TCCTCTGTAAGCCATCCTGC

csrB2-CF	CCGGAATTC ACCTGTAGACGGAACTACAGAT
csrB2-CR	CCC CTCGAG ATCATCCATAGAAGAAAGGGCG
LacZ reporter	
pub-F	GGATCCTTTTATCGCTGACTCGCAGT
pub-R	AAGCTTGTAATCATGGTCATTTACGCCTCCTTAAATTCCTG
ssoR-F	GAATTCCGCGTTCGTAACAACTTCAG
ssoR-R	AAGCTTGTAATCATGGTCATGATCATCATCGACTAACAGTATTCTA
P*arcA*-F	GAATTCCCGGTGCTAGCCGC
P*arcA*-R	AAGCTTGTAATCATGGTCATGTGCGGATTTTGCATATTAAGTACCTA
Site-directed mutagenesis	
$SsoR^{D52N}$-F	CGCCCGATATCGTTATGCTCAATCTGGTAATGCCGCAAATG
$SsoR^{D52N}$-R	CAAAAGAGCGGATGCATGTAAGC
$SsoR^{D52E}$-F	CCCGATATCGTTATGCTCGAACTGGTAATGCCGCAAATGGA
$SsoR^{D52E}$-R	CAAAAGAGCGGATGCATGTAAGC
Protein expression	
SsoR-F	CATATGATGAGTAGAATACTGTTAGTCGAT
SsoR-R	CTCGAGTTAAGCGCTAAAACTGTATCC
Fur-F	CATATGATGACAGATGGAAATCAAGCG
Fur-R	CTCGAGTTATTCGTCGTTGTGCTCG
qRT-PCR	
pubA-qF	CAAACACCTTTTATGTCCGATTTG
pubA-qR	AGGAACAGGCACAACGCCCTATC
csrB1-qF	AGCTTAAGGATTAAGCATGACTCG
csrB1-qR	CCTGCTCAATACCTTCCATAGACG
csrB2-qF	GGGATAGACTATCACGGATAGATAGC
csrB2-qR	CATCATCCGTGAAATAGGTTCAATTG
EMSA	
pub-F	CCGTATTTCAGCTCCTTATTTACC
pub-R	AGTACGACTCATCCGACAAC
ssoR-F	CATGTATCTGATAACACCACTGTTAG
ssoR-R	TACGTTACTAACGGTAGTGACG

Description of Supplementary Movie 1

The video depicts the 180-280 ns trajectory of $VbrR^{D-RD-D51N}$, showing the switch residue as it transitions through four states. The REC domain was represented in green cartoon form, while the switch residue was illustrated as cyan sticks, and T99 was shown in cyan lines, with the α4-helix concealed. Furthermore, the distance between the CZ atom of the switch residue and the N atom in T99's backbone, as well as the diameter of the benzene ring, were measured. The transition between intermediate state A and intermediate state B, as well as from intermediate state B to the outer state, is accompanied by the compression of the benzene ring of the switching molecule. Moreover, the benzene ring is compressed and flipped when in the outer state, suggesting that this state becomes unstable after dimerization.

Supplementary References:

1. Lassak, J., Henche, A.L., Binnenkade, L. & Thormann, K.M. ArcS, the cognate sensor kinase in an atypical Arc system of *Shewanella oneidensis* MR-1. *Appl. Environ. Microbiol.* **76**, 3263-3274 (2010).
2. Bordi, C., *et al.* Genes regulated by TorR, the trimethylamine oxide response regulator of *Shewanella oneidensis*. *J. Bacteriol.* **186**, 4502-4509 (2004).
3. Binnenkade, L., Lassak, J. & Thormann, K.M. Analysis of the BarA/UvrY two-component system in *Shewanella oneidensis* MR-1. *PLoS One* **6**, e23440 (2011).
4. Leaphart, A.B., *et al.* Transcriptome profiling of *Shewanella oneidensis* gene expression following exposure to acidic and alkaline pH. *J. Bacteriol.* **188**, 1633-1642 (2006).
5. Bouillet, S., *et al.* The general stress response σ^S is regulated by a partner switch in the gram-negative bacterium *Shewanella oneidensis*. *J. Biol. Chem.* **291**, 26151-26163 (2016).
6. Bouillet, S., Genest, O., Méjean, V. & Iobbi-Nivol, C. Protection of the general stress response σ^S factor by the CrsR regulator allows a rapid and efficient adaptation of *Shewanella oneidensis*. *J. Biol. Chem.* **292**, 14921-14928 (2017).
7. Yin, J., *et al.* Development of whole-cell biosensors for screening of peptidoglycan-targeting antibiotics in a gram-negative bacterium. *Appl. Environ. Microbiol.* **88**, e0084622 (2022).
8. Plate, L. & Marletta, M.A. Phosphorylation-dependent derepression by the response regulator HnoC in the *Shewanella oneidensis* nitric oxide signaling network. *Proc Natl Acad Sci USA* **110**, E4648-E4657 (2013).
9. Nisbett, L.-M., *et al.* NosP signaling modulates the NO/H-NOX-mediated multicomponent c-di-gmp network and biofilm formation in *Shewanella oneidensis*. *Biochemistry* **58**, 4827-4841 (2019).
10. Rao, M., Herzik, M.A., Iavarone, A.T. & Marletta, M.A. Nitric oxide-induced conformational changes govern h-nox and histidine kinase interaction and regulation in *Shewanella oneidensis*. *Biochemistry* **56**, 1274-1284 (2017).
11. Deutschbauer, A., *et al.* Evidence-based annotation of gene function in *Shewanella oneidensis* MR-1 using genome-wide fitness profiling across 121 conditions. *PLos Genet.* **7**, e1002385 (2011).
12. Gao, T., Shi, M. & Gao, H. Partially reciprocal replacement of FlrA and FlrC in regulation of *Shewanella oneidensis* flagellar biosynthesis. *J. Bacteriol.* **200**(2018).
13. Dong, Y., *et al.* A Crp-dependent two-component system regulates nitrate and nitrite respiration in *Shewanella oneidensis*. *PLoS One* **7**, e51643 (2012).
14. Yang, Y., *et al.* Snapshot of iron response in *Shewanella oneidensis* by gene network reconstruction. *BMC Genomics* **10**, 131 (2009).
15. Le Laz, S., *et al.* Expression of terminal oxidases under nutrient-starved conditions in *Shewanella oneidensis*: detection of the A-type cytochrome c oxidase. *Sci. Rep.* **6**, 19726 (2016).
16. Schmidl, S.R., *et al.* Rewiring bacterial two-component systems by modular DNA-binding domain swapping. *Nat. Chem. Biol.* **15**, 690-698 (2019).
17. Ikegami, A., Nakasone, K., Kato, C., Usami, R. & Horikoshi, K. Structural analysis of the *ntrBC* genes of deep-sea piezophilic *Shewanella violacea*. *Biosci. Biotechnol. Biochem.* **64**, 915-918 (2000).
18. Brown, S.D., *et al.* Cellular response of *Shewanella oneidensis* to strontium stress. *Appl. Environ. Microbiol.* **72**, 890-900 (2006).
19. Yuan, J., Wei, B., Shi, M. & Gao, H. Functional assessment of EnvZ/OmpR two-component system in *Shewanella oneidensis*. *PLoS One* **6**, e23701 (2011).
20. Dong, Z., Guo, S., Fu, H. & Gao, H. Investigation of a spontaneous mutant reveals novel features of iron uptake in *Shewanella oneidensis*. *Sci. Rep.* **7**, 11788 (2017).
21. Fu, H., Liu, L., Dong, Z., Guo, S. & Gao, H. Dissociation between iron and heme biosyntheses is largely accountable for respiration defects of *Shewanella oneidensis fur* mutants. *Appl. Environ. Microbiol.* **84**, e00039-00018 (2018).
22. Jin, M., *et al.* Unique organizational and functional features of the cytochrome *c* maturation system in *Shewanella oneidensis*. *PLoS One* **8**, e75610 (2013).
23. Fu, H., *et al.* Crp-dependent cytochrome *bd* oxidase confers nitrite resistance to *Shewanella oneidensis*. *Environ. Microbiol.* **15**, 2198-2212 (2013).
24. Meng, Q., Sun, Y. & Gao, H. Cytochromes *c* constitute a layer of protection against nitric oxide but not nitrite. *Appl. Environ. Microbiol.* **84**, e01255-01218 (2018).
25. Wu, L., Wang, J., Tang, P., Chen, H. & Gao, H. Genetic and molecular characterization of flagellar assembly in *Shewanella oneidensis*. *PLoS One* **6**, e21479 (2011).
