# Supplementary material for: Multifaceted regulation of siderophore synthesis by multiple regulatory systems in Shewanella oneidensis
Source: Commun Biol. 2024 Apr 25;7:498. doi: 10.1038/s42003-024-06193-7 (PMC11045786; doi:10.1038/s42003-024-06193-7)
Supplement: Supplementary file 3 — Description of Additional Supplementary Files [file 42003_2024_6193_MOESM3_ESM.pdf]

Description of Additional Supplementary Files

File name: Supplementary Data
Description: The source data behind the graphs in the paper

File name: Supplementary Movie
Description: The video depicts the 180-280 ns trajectory of VbrRD-RD-D51N, showing the switch residue as it transitions through four states.
